# Supplementary material for: Prokaryotic responses to a warm temperature anomaly in northeast subarctic Pacific waters
Source: Commun Biol. 2021 Oct 22;4:1217. doi: 10.1038/s42003-021-02731-9 (PMC8536700; doi:10.1038/s42003-021-02731-9)
Supplement: Supplementary file 2 — Supplementary Information [file 42003_2021_2731_MOESM2_ESM.pdf]

Supplementary Information For:

Prokaryotic response to a warm water temperature anomaly in northeast subarctic Pacific waters

Sachia J. Traving[1†], Colleen T. E. Kellogg[2], Tetjana Ross[3], Ryan McLaughlin[4], Brandon Kieft[1], Grace Y. Ho[1‡], Angelica Pena[3], Martin Krzywinski[5], Marie Robert[3], Steven J. Hallam[1,4,6,7,8*]

Affiliations
[1]Department of Microbiology & Immunology, University of British Columbia, Vancouver, BC V6T 1Z1, Canada.
[2]Hakai Institute, BC, Canada.
[3]Institute of Ocean Sciences, Fisheries and Ocean Canada, BC, Canada.
[4]Graduate Program in Bioinformatics, University of British Columbia, Vancouver, BC V6T 1Z4, Canada.
[5]Genome Sciences Centre, BC Cancer Agency, Vancouver, BC, V5Z 4S6, Canada
[6]Genome Science and Technology Program, University of British Columbia, 2329 West Mall, Vancouver, BC V6T 1Z4, Canada.
[7]Life Sciences Institute, University of British Columbia, Vancouver, BC V6T 1Z3, Canada.
[8]ECOSCOPE Training Program, University of British Columbia, Vancouver, BC V6T 1Z3, Canada.

[†]Current address: HADAL and Nordcee, Department of Biology, University of Southern Denmark, Campusvej 55, 5230 Odense M, Denmark

[‡]Current address: Max Planck Institute for Marine Microbiology, Celsiusstraße 1, 28359 Bremen, Germany

[*]To whom correspondence should be addressed:
University of British Columbia, Department of Microbiology and Immunology
2552-2350 Health Sciences Mall, Vancouver, BC Canada V6T 1Z3
Office: (604) 827-3420 email: shallam@mail.ubc.ca

Supplementary Tables

Table S1. Network and indicator analysis statistics and taxonomy for OTU indicators found for the 10-100 m of the water column.

OTU ID	Network Conn.	Indicator Value	p-value	Blob Ind.?	Phylum (mod)	Class	Order	Family	Genus	Species	Ave. % Abund.
OTU_659	4	0.713	0.006	Yes	Actinobacteria	Acidimicrobiia	Microtrichales	Microtrichaceae	Sva0996 marine group		0.01816
OTU_9	5	0.767	0.001	Yes	Alphaproteobacteria	Alphaproteobacteria	SAR11 clade	Clade II			3.87279
OTU_1076	10	0.739	0.009	Yes	Alphaproteobacteria	Alphaproteobacteria	uncultured				0.02322
OTU_265	8	0.774	0.01	Yes	Alphaproteobacteria	Alphaproteobacteria	SAR11 clade	Clade II			0.01501
OTU_363	10	0.813	0.016	Yes	Alphaproteobacteria	Alphaproteobacteria	SAR11 clade	Clade I	uncultured		0.02582
OTU_801	6	0.77	0.009	Yes	Alphaproteobacteria	Alphaproteobacteria	SAR11 clade				0.00482
OTU_603	34	0.841	0.004	Yes	Alphaproteobacteria	Alphaproteobacteria	SAR11 clade	Clade I	Clade Ib		0.01086
OTU_699	8	0.768	0.001	Yes	Alphaproteobacteria	Alphaproteobacteria	SAR11 clade	Clade II			0.12093
OTU_417	36	0.758	0.009	Yes	Alphaproteobacteria	Alphaproteobacteria	SAR11 clade	Clade II			0.01644
OTU_228	9	0.858	0.001	Yes	Alphaproteobacteria	Alphaproteobacteria	Rhodobacterales	Rhodobacteraceae			0.18397
OTU_81	13	0.721	0.042	Yes	Bacteroidetes	Bacteroidia	Cytophagales	Cyclobacteriaceae	Marinoscillum	Ambiguous_taxa	0.13865
OTU_917	4	0.869	0.001	Yes	Bacteroidetes	Bacteroidia	Cytophagales	Cyclobacteriaceae	Marinoscillum	Ambiguous_taxa	0.06836
OTU_12	14	0.906	0.012	Yes	Bacteroidetes	Bacteroidia	Flavobacteriales	Flavobacteriaceae	NS2b marine group		0.05550
OTU_135	6	0.816	0.006	Yes	Bacteroidetes	Bacteroidia	Flavobacteriales	NS9 marine group			0.45522
OTU_94	11	0.763	0.04	Yes	Dadabacteria	Dadabacteriia	Dadabacteriales	uncultured marine bacterium	uncultured marine bacterium	uncultured marine bacterium	0.23413
OTU_180	4	0.852	0.001	Yes	Euryarchaeota	Thermoplasmata	Marine Group III	Ambiguous_taxa	Ambiguous_taxa	Ambiguous_taxa	0.18288
OTU_147	39	0.802	0.002	Yes	Euryarchaeota	Thermoplasmata	Marine Group II				0.00846
OTU_246	4	0.849	0.001	Yes	Euryarchaeota	Thermoplasmata	Marine Group II				0.04239
OTU_1332	22	0.725	0.002	Yes	Euryarchaeota	Thermoplasmata	Marine Group II	Ambiguous_taxa	Ambiguous_taxa	Ambiguous_taxa	0.00816
OTU_200	4	0.742	0.006	Yes	Gammaproteobacteria	Gammaproteobacteria	SAR86 clade				0.57320
OTU_545	6	0.973	0.001	Yes	Gammaproteobacteria	Gammaproteobacteria	Oceanospirillales	Pseudohongiellaceae	Pseudohongiella		0.08717
OTU_373	4	0.825	0.001	Yes	Gammaproteobacteria	Gammaproteobacteria	HOC36	Ambiguous_taxa	Ambiguous_taxa	Ambiguous_taxa	0.03844
OTU_153	30	0.795	0.008	Yes	Gammaproteobacteria	Gammaproteobacteria	Ectothiorhodospirales	Ectothiorhodospiraceae	uncultured		0.00861
OTU_1411	4	0.775	0.003	Yes	Gammaproteobacteria	Gammaproteobacteria	Oceanospirillales	Pseudohongiellaceae	Pseudohongiella		0.02659
OTU_83	15	0.755	0.038	Yes	Gammaproteobacteria	Gammaproteobacteria	SAR86 clade	Ambiguous_taxa	Ambiguous_taxa	Ambiguous_taxa	0.92118
OTU_812	17	0.756	0.001	Yes	Gammaproteobacteria	Gammaproteobacteria	SAR86 clade	uncultured gamma proteobacterium	uncultured gamma proteobacterium	uncultured gamma proteobacterium	0.00892
OTU_485	6	0.909	0.001	Yes	Margulisbacteria	uncultured marine cyanobacterium	uncultured marine cyanobacterium	uncultured marine cyanobacterium	uncultured marine cyanobacterium	uncultured marine cyanobacterium	0.04413

OTU_372	12	0.744	0.01	Yes	Marinimicrobia (SAR406 clade)						0.00963
OTU_676	5	0.734	0.001	Yes	Marinimicrobia (SAR406 clade)	metagenome	metagenome	metagenome	metagenome	metagenome	0.00455
OTU_179	13	0.719	0.03	Yes	Marinimicrobia (SAR406 clade)	Ambiguous_taxa	Ambiguous_taxa	Ambiguous_taxa	Ambiguous_taxa	Ambiguous_taxa	0.00404
OTU_111	37	0.732	0.027	Yes	Marinimicrobia (SAR406 clade)	Ambiguous_taxa	Ambiguous_taxa	Ambiguous_taxa	Ambiguous_taxa	Ambiguous_taxa	0.00925
OTU_148	16	0.724	0.02	Yes	Marinimicrobia (SAR406 clade)						0.04585
OTU_54	13	0.854	0.005	Yes	Marinimicrobia (SAR406 clade)						0.04103
OTU_116	8	0.853	0.001	Yes	Planctomycetes	Planctomycetacia	Pirellulales	Pirellulaceae	Rhodopirellula	uncultured Planctomycetaceae bacterium	0.01320
OTU_275	23	0.714	0.007	Yes	Verrucomicrobia	Verrucomicrobiae	Opitutales	Puniceicoccaceae	MB11C04 marine group		0.00728
OTU_1035	6	0.765	0.022	No	Alphaproteobacteria	Alphaproteobacteria	Rhodospirillales	AEGEAN-169 marine group			0.12145
OTU_205	7	0.793	0.005	No	Alphaproteobacteria	Alphaproteobacteria	Rhodospirillales	Magnetospiraceae	uncultured	Ambiguous_taxa	0.05113
OTU_67	15	0.798	0.01	No	Alphaproteobacteria	Alphaproteobacteria	Rhodobacterales	Rhodobacteraceae			0.44236
OTU_855	10	0.717	0.036	No	Alphaproteobacteria	Alphaproteobacteria	Rhodospirillales	Magnetospiraceae	uncultured	uncultured alpha proteobacterium	0.00772
OTU_4	5	0.748	0.005	No	Alphaproteobacteria	Alphaproteobacteria	SAR11 clade	Clade I	Clade Ia		14.54660
OTU_471	9	0.738	0.017	No	Deltaproteobacteria	Deltaproteobacteria	NB1-j	uncultured marine bacterium	uncultured marine bacterium	uncultured marine bacterium	0.01120
OTU_1148	40	0.817	0.001	No	Deltaproteobacteria	Deltaproteobacteria	SAR324 clade (Marine group B)	uncultured marine bacterium	uncultured marine bacterium	uncultured marine bacterium	0.11613
OTU_948	7	0.749	0.034	No	Gammaproteobacteria	Gammaproteobacteria	SAR86 clade	Ambiguous_taxa	Ambiguous_taxa	Ambiguous_taxa	0.01099
OTU_1037	5	0.788	0.001	No	Gammaproteobacteria	Gammaproteobacteria	Tenderiales	Tenderiaceae	Candidatus Tenderia	uncultured bacterium	0.01088
OTU_1323	7	0.754	0.009	No	Gammaproteobacteria	Gammaproteobacteria	Betaproteobacteriales	Methylophilaceae	OM43 clade	Ambiguous_taxa	0.13965
OTU_62	16	0.835	0.011	No	Gammaproteobacteria	Gammaproteobacteria	Oceanospirillales	Pseudohongiellaceae	Pseudohongiella		0.18021
OTU_199	8	0.768	0.018	No	Gammaproteobacteria	Gammaproteobacteria	Ectothiorhodospirales	Ectothiorhodospiraceae	uncultured	Ambiguous_taxa	0.35610
OTU_215	14	0.811	0.002	No	Gammaproteobacteria	Gammaproteobacteria	Oceanospirillales	Pseudohongiellaceae	Pseudohongiella		0.24941
OTU_441	7	0.761	0.007	No	Gammaproteobacteria	Gammaproteobacteria	Cellvibrionales	Halieaceae	OM60(NOR5) clade		0.01619
OTU_942	5	0.911	0.001	No	Planctomycetes	Phycisphaerae	Phycisphaerales	Phycisphaeraceae	CL500-3	uncultured marine bacterium	0.00886
OTU_739	11	0.76	0.012	No	Planctomycetes	Phycisphaerae	Phycisphaerales	Phycisphaeraceae	CL500-3	uncultured Phycisphaeraceae bacterium	0.00968

Table S2. Network and indicator analysis statistics and taxonomy for OTU indicators found for the 150-200 m of the water column.

OTU ID	Network Conn.	Indicator Value	p-value	Blob Ind.?	Phylum (mod)	Class	Order	Family	Genus	Species	Ave. % Abund.
OTU_558	4	0.796	0.048	Yes	Acidobacteria	Subgroup 21	Ambiguous_taxa	Ambiguous_taxa	Ambiguous_taxa	Ambiguous_taxa	0.00218
OTU_1390	5	0.786	0.007	Yes	Alphaproteobacteria	Alphaproteobacteria	SAR11 clade	Clade II			0.01221
OTU_664	16	0.765	0.014	Yes	Alphaproteobacteria	Alphaproteobacteria	SAR11 clade	Clade I	Clade Ib		0.14864
OTU_1310	5	0.824	0.01	Yes	Alphaproteobacteria	Alphaproteobacteria	SAR11 clade				0.00158
OTU_1252	16	0.827	0.005	Yes	Alphaproteobacteria	Alphaproteobacteria	SAR11 clade	Clade II			0.01549
OTU_363	11	0.794	0.004	Yes	Alphaproteobacteria	Alphaproteobacteria	SAR11 clade	Clade I	uncultured		0.20285
OTU_808	4	0.823	0.008	Yes	Alphaproteobacteria	Alphaproteobacteria	SAR11 clade				0.00330
OTU_325	16	0.742	0.039	Yes	Alphaproteobacteria	Alphaproteobacteria	SAR11 clade	Clade II			0.18057
OTU_486	22	0.749	0.001	Yes	Alphaproteobacteria	Alphaproteobacteria	SAR11 clade	Clade II			0.10533
OTU_143	26	0.736	0.024	Yes	Alphaproteobacteria	Alphaproteobacteria	SAR11 clade	Clade II			0.60792
OTU_15	17	0.805	0.003	Yes	Alphaproteobacteria	Alphaproteobacteria	SAR11 clade				0.02882
OTU_603	14	0.785	0.001	Yes	Alphaproteobacteria	Alphaproteobacteria	SAR11 clade	Clade I	Clade Ib		0.19806
OTU_1022	6	0.845	0.007	Yes	Alphaproteobacteria	Alphaproteobacteria	SAR11 clade				0.00475
OTU_38	23	0.758	0.004	Yes	Alphaproteobacteria	Alphaproteobacteria	SAR11 clade	Clade I	Clade Ib		2.30978
OTU_711	10	0.79	0.004	Yes	Alphaproteobacteria	Alphaproteobacteria	SAR11 clade	Clade II			0.01044
OTU_661	9	0.786	0.004	Yes	Alphaproteobacteria	Alphaproteobacteria	SAR11 clade	uncultured	uncultured bacterium	uncultured bacterium	0.02780
OTU_460	5	0.84	0.002	Yes	Alphaproteobacteria	Alphaproteobacteria	Rhodospirillales	AEGEAN-169 marine group	uncultured bacterium	uncultured bacterium	0.00109
OTU_41	29	0.747	0.002	Yes	Alphaproteobacteria	Alphaproteobacteria	SAR11 clade	Clade II			1.11197
OTU_197	13	0.878	0.004	Yes	Alphaproteobacteria	Alphaproteobacteria	Parvibaculales	OCS116 clade	uncultured bacterium	uncultured bacterium	0.00354
OTU_103	25	0.764	0.001	Yes	Alphaproteobacteria	Alphaproteobacteria	SAR11 clade				0.17511
OTU_311	7	0.824	0.013	Yes	Alphaproteobacteria	Alphaproteobacteria	SAR11 clade	Clade I	uncultured		0.01088
OTU_182	19	0.771	0.009	Yes	Alphaproteobacteria	Alphaproteobacteria	SAR11 clade	Clade II			0.18446
OTU_23	26	0.765	0.003	Yes	Alphaproteobacteria	Alphaproteobacteria	SAR11 clade	Clade I			4.92850
OTU_430	9	0.863	0.003	Yes	Chloroflexi	Dehalococcoidia	SAR202 clade	Ambiguous_taxa	Ambiguous_taxa	Ambiguous_taxa	0.01175
OTU_255	9	0.859	0.003	Yes	Chloroflexi	Dehalococcoidia	SAR202 clade	Ambiguous_taxa	Ambiguous_taxa	Ambiguous_taxa	0.00423
OTU_1016	11	0.815	0.022	Yes	Chloroflexi	Dehalococcoidia	SAR202 clade				0.00370
OTU_108	23	0.749	0.025	Yes	Gammaproteobacteria	Gammaproteobacteria					0.41552
OTU_71	7	0.858	0.001	Yes	Gammaproteobacteria	Gammaproteobacteria	Oceanospirillales	Pseudohongiellaceae	Pseudohongiella		0.01931
OTU_224	15	0.844	0.005	Yes	Gammaproteobacteria	Gammaproteobacteria	UBA10353 marine group	Ambiguous_taxa	Ambiguous_taxa	Ambiguous_taxa	0.00860
OTU_727	7	0.884	0.002	Yes	Gammaproteobacteria	Gammaproteobacteria	SAR86 clade				0.00169
OTU_400	11	0.881	0.004	Yes	Gammaproteobacteria	Gammaproteobacteria					0.00857
OTU_33	11	0.829	0.001	Yes	Gammaproteobacteria	Gammaproteobacteria	SAR86 clade	marine metagenome	marine metagenome	marine metagenome	0.19306
OTU_1327	7	0.892	0.001	Yes	Gammaproteobacteria	Gammaproteobacteria	SAR86 clade	uncultured gamma proteobacterium HF4000_19M20	uncultured gamma proteobacterium HF4000_19M20	uncultured gamma proteobacterium HF4000_19M20	0.00130
OTU_144	12	0.817	0.034	Yes	Gammaproteobacteria	Gammaproteobacteria	HOC36	Ambiguous_taxa	Ambiguous_taxa	Ambiguous_taxa	0.01501

OTU_1398	9	0.866	0.007	Yes	Gammaproteobacteria	Gammaproteobacteria	pltb-vmat-80	uncultured bacterium	uncultured bacterium	uncultured bacterium	0.00132
OTU_1183	17	0.796	0.008	Yes	Marinimicrobia (SAR406 clade)						0.00084
OTU_408	10	0.843	0.002	Yes	Marinimicrobia (SAR406 clade)						0.00096
OTU_90	16	0.871	0.004	Yes	Marinimicrobia (SAR406 clade)	Ambiguous_taxa	Ambiguous_taxa	Ambiguous_taxa	Ambiguous_taxa	Ambiguous_taxa	0.02154
OTU_34	19	0.831	0.004	Yes	Marinimicrobia (SAR406 clade)	Ambiguous_taxa	Ambiguous_taxa	Ambiguous_taxa	Ambiguous_taxa	Ambiguous_taxa	0.11647
OTU_190	15	0.846	0.017	Yes	Marinimicrobia (SAR406 clade)	Ambiguous_taxa	Ambiguous_taxa	Ambiguous_taxa	Ambiguous_taxa	Ambiguous_taxa	0.00758
OTU_644	9	0.781	0.028	Yes	Nitrospinae	Nitrospinia	Nitrospinales	Nitrospinaceae	Nitrospina		0.01617
OTU_532	9	0.83	0.02	Yes	Planctomycetes	028H05-P-BN-P5	Ambiguous_taxa	Ambiguous_taxa	Ambiguous_taxa	Ambiguous_taxa	0.02303
OTU_305	12	0.798	0.018	Yes	Thaumarchaeota	Nitrososphaeria	Nitrosopumilales	Nitrosopumilaceae	uncultured marine archaeon	uncultured marine archaeon	0.22275
OTU_142	18	0.792	0.014	Yes	Thaumarchaeota	Nitrososphaeria	Nitrosopumilales	Nitrosopumilaceae	uncultured marine archaeon	uncultured marine archaeon	0.11405
OTU_7	22	0.826	0.01	Yes	Thaumarchaeota	Nitrososphaeria	Nitrosopumilales	Nitrosopumilaceae	uncultured thaumarchaeote	uncultured thaumarchaeote	0.40146
OTU_50	5	0.812	0.001	No	Alphaproteobacteria	Alphaproteobacteria	Rhodobacterales	Rhodobacteraceae	Ascidiaceihabitans		0.06122
OTU_1241	9	0.829	0.015	No	Alphaproteobacteria	Alphaproteobacteria	Sneathiellales	Sneathiellaceae			0.00494
OTU_438	4	0.853	0.01	No	Alphaproteobacteria	Alphaproteobacteria	SAR11 clade	Clade I	Clade Ia	uncultured Pelagibacterales bacterium	0.26710
OTU_67	20	0.771	0.001	No	Alphaproteobacteria	Alphaproteobacteria	Rhodobacterales	Rhodobacteraceae			0.27414
OTU_269	18	0.911	0.015	No	Alphaproteobacteria	Alphaproteobacteria	Rhodovibrionales	Kiloniellaceae	uncultured	Ambiguous_taxa	0.02288
OTU_428	17	0.904	0.03	No	Alphaproteobacteria	Alphaproteobacteria	uncultured				0.01149
OTU_22	31	0.921	0.04	No	Alphaproteobacteria	Alphaproteobacteria	Parvibaculales	OCS116 clade			0.03348
OTU_754	16	0.804	0.001	No	Alphaproteobacteria	Alphaproteobacteria	Rhodospirillales	Magnetospiraceae	uncultured	uncultured alpha proteobacterium	0.02494
OTU_935	15	0.885	0.032	No	Alphaproteobacteria	Alphaproteobacteria	Rhodospirillales	Magnetospiraceae	uncultured	Ambiguous_taxa	0.00955
OTU_107	34	0.95	0.019	No	Alphaproteobacteria	Alphaproteobacteria	Rhodobacterales	Rhodobacteraceae	Lentibacter	uncultured bacterium	0.02289
OTU_105	4	0.786	0.008	No	Bacteroidetes	Bacteroidia	Flavobacteriales	NS9 marine group	Ambiguous_taxa	Ambiguous_taxa	0.31213
OTU_56	17	0.767	0.006	No	Bacteroidetes	Bacteroidia	Flavobacteriales	NS9 marine group			1.00280
OTU_977	10	0.873	0.023	No	Bacteroidetes	Bacteroidia	Flavobacteriales	NS9 marine group			0.00617
OTU_78	9	0.765	0.034	No	Bacteroidetes	Bacteroidia	Flavobacteriales	NS9 marine group			0.27158
OTU_135	16	0.753	0.035	No	Bacteroidetes	Bacteroidia	Flavobacteriales	NS9 marine group			0.24386
OTU_12	32	0.738	0.023	No	Bacteroidetes	Bacteroidia	Flavobacteriales	Flavobacteriaceae	NS2b marine group		0.93282
OTU_261	18	0.847	0.015	No	Chloroflexi	Dehalococcoidia	SAR202 clade	Ambiguous_taxa	Ambiguous_taxa	Ambiguous_taxa	0.08453
OTU_406	17	0.883	0.025	No	Chloroflexi	Dehalococcoidia	SAR202 clade	Ambiguous_taxa	Ambiguous_taxa	Ambiguous_taxa	0.00890
OTU_330	12	0.79	0.031	No	Chloroflexi	Dehalococcoidia	SAR202 clade				0.07371
OTU_1407	4	0.873	0.031	No	Chloroflexi	Dehalococcoidia	SAR202 clade	uncultured Chloroflexi bacterium	uncultured Chloroflexi bacterium	uncultured Chloroflexi bacterium	0.00985
OTU_94	26	0.866	0.025	No	Dadabacteria	Dadabacteriia	Dadabacteriales	uncultured marine bacterium	uncultured marine bacterium	uncultured marine bacterium	0.07169
OTU_19	4	0.794	0.008	No	Euryarchaeota	Thermoplasmata	Marine Group II	uncultured marine archaeon	uncultured marine archaeon	uncultured marine archaeon	0.42841
OTU_342	7	0.909	0.001	No	Gammaproteobacteria	Gammaproteobacteria	Cellvibrionales	Porticoccaceae	SAR92 clade	Ambiguous_taxa	0.01249
OTU_519	14	0.88	0.028	No	Gammaproteobacteria	Gammaproteobacteria	Oceanospirillales	Nitrincolaceae	uncultured	uncultured marine bacterium	0.00439

OTU_218	14	0.871	0.018	No	Gammaproteobacteria	Gammaproteobacteria	Ga0077536	Ambiguous_taxa	Ambiguous_taxa	Ambiguous_taxa	0.00771
OTU_40	30	0.923	0.049	No	Gammaproteobacteria	Gammaproteobacteria	SAR86 clade	uncultured SAR86 cluster bacterium	uncultured SAR86 cluster bacterium	uncultured SAR86 cluster bacterium	0.02485
OTU_672	28	0.788	0.036	No	Gammaproteobacteria	Gammaproteobacteria	OM182 clade	uncultured gamma proteobacterium	uncultured gamma proteobacterium	uncultured gamma proteobacterium	0.00433
OTU_199	4	0.846	0.016	No	Gammaproteobacteria	Gammaproteobacteria	Ectothiorhodospirales	Ectothiorhodospiraceae	uncultured	Ambiguous_taxa	0.02889
OTU_1108	12	0.778	0.011	No	Gammaproteobacteria	Gammaproteobacteria	SAR86 clade	uncultured gamma proteobacterium	uncultured gamma proteobacterium	uncultured gamma proteobacterium	0.04297
OTU_810	10	0.927	0.028	No	Gemmatimonadetes	BD2-11 terrestrial group	uncultured Gemmatimonadetes bacterium	uncultured Gemmatimonadetes bacterium	uncultured Gemmatimonadetes bacterium	uncultured Gemmatimonadetes bacterium	0.00863
OTU_676	7	0.805	0.001	No	Marinimicrobia (SAR406 clade)	metagenome	metagenome	metagenome	metagenome	metagenome	0.03006
OTU_86	17	0.762	0.004	No	Nitrospinae	Nitrospinia	Nitrospinales	Nitrospinaceae	Nitrospina		0.75770
OTU_514	19	0.833	0.033	No	Nitrospinae	Nitrospinia	Nitrospinales	Nitrospinaceae	LS-NOB	uncultured delta proteobacterium	0.03729
OTU_267	39	0.895	0.032	No	Nitrospinae	Nitrospinia	Nitrospinales	Nitrospinaceae	LS-NOB	uncultured delta proteobacterium	0.06653
OTU_1328	13	0.867	0.001	No	PAUC34f	uncultured marine bacterium	uncultured marine bacterium	uncultured marine bacterium	uncultured marine bacterium	uncultured marine bacterium	0.01098
OTU_556	11	0.774	0.028	No	Planctomycetes	Phycisphaerae	Phycisphaerales	Phycisphaeraceae	FS140-16B-02 marine group	uncultured bacterium	0.01552
OTU_945	9	0.848	0.047	No	Planctomycetes	Phycisphaerae	MSBL9	SM23-30	uncultured bacterium	uncultured bacterium	0.01630
OTU_882	7	0.854	0.027	No	Verrucomicrobia	Verrucomicrobiae	Opitutales	Puniceicoccaceae	MB11C04 marine group	Ambiguous_taxa	0.00797

Supplementary Figures

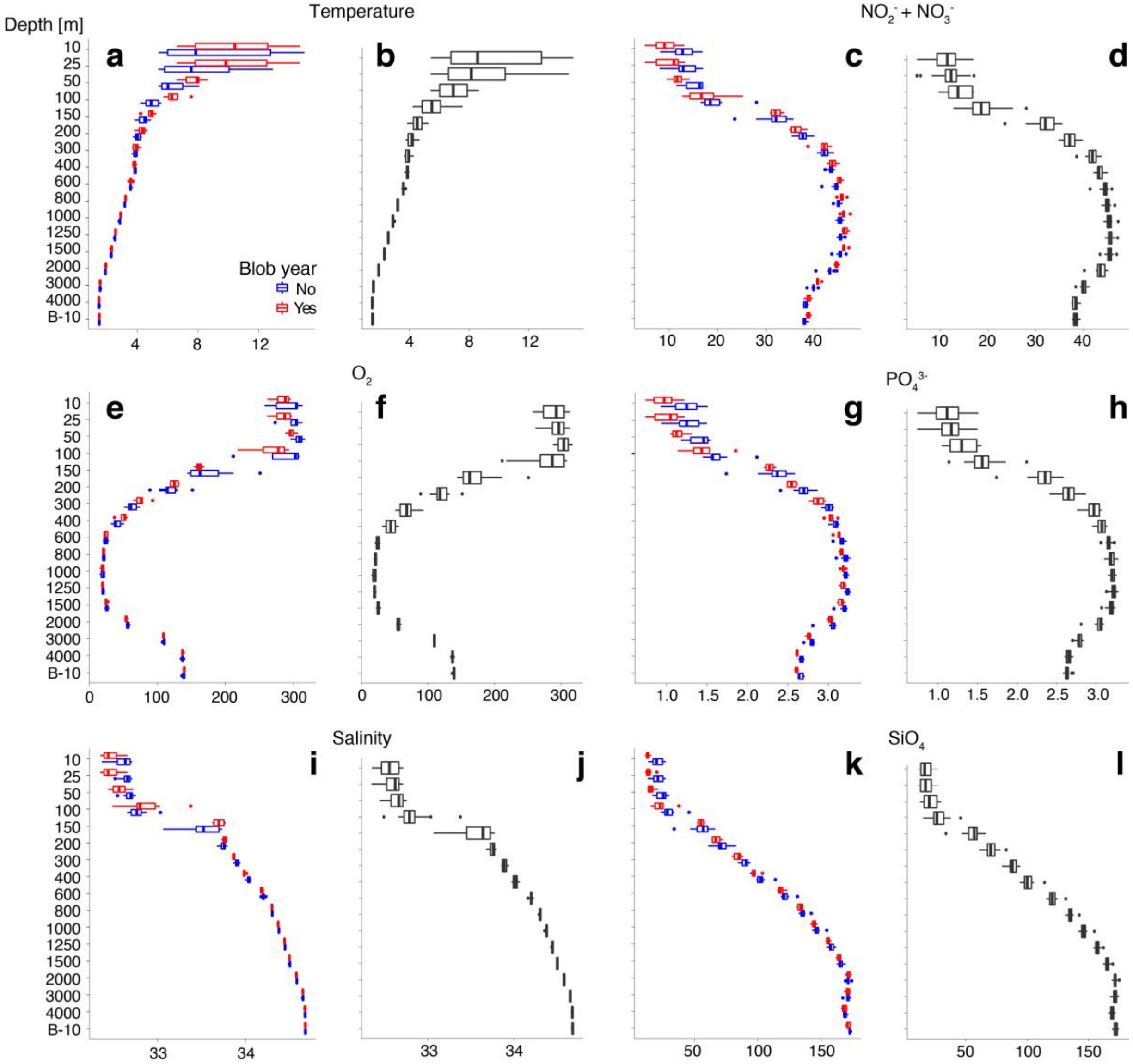

Figure S1. Boxplots of environmental parameters measured by IOS, Canada as part of the Line P program. Total concentration and partitioned after Blob and non-Blob years, for a,b) temperature, c,d) NO_2^- and NO_3^- [µmol L^{-1}], e,f) O_2 [µm kg^{-1}], g,h) PO_4^{3-} [µmol L^{-1}], i,j) salinity, and k,l) SiO_4 [µmol L^{-1}]. The centre line of the boxplot represents the median, the lower box limit the 25% quartile, the upper box limit the 75% quartile, and the whiskers extend to 1.5 * IQR.

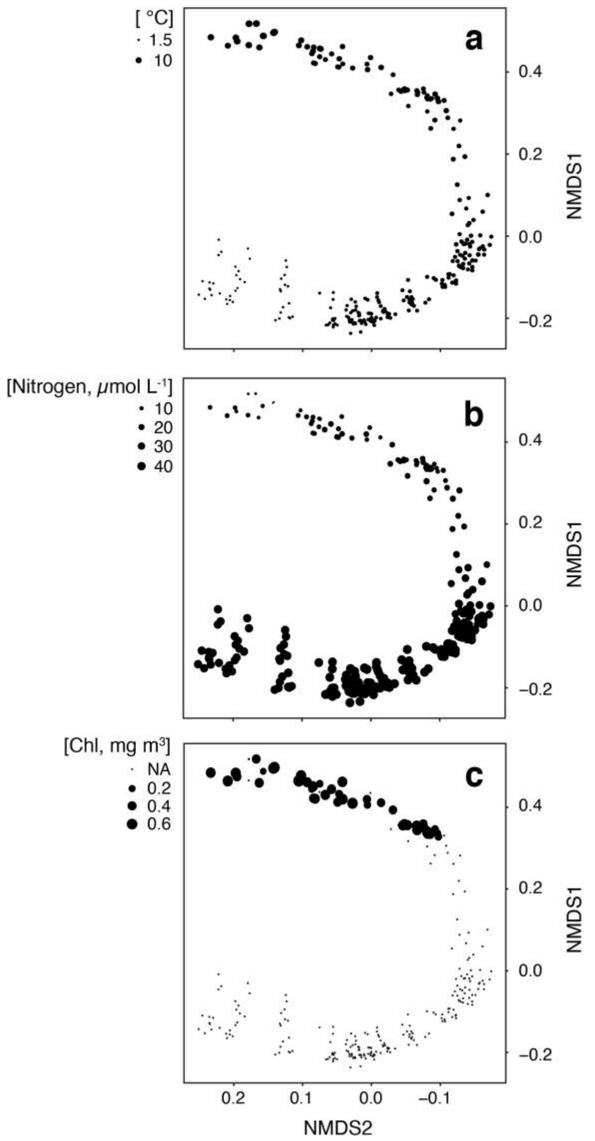

Figure S2. Bray-Curtis dissimilarity of all samples in a non-metric multidimensional scaling (nMDS) plot weighted by a) temperature, b) nitrogen concentration, and c) total Chl *a* concentration.

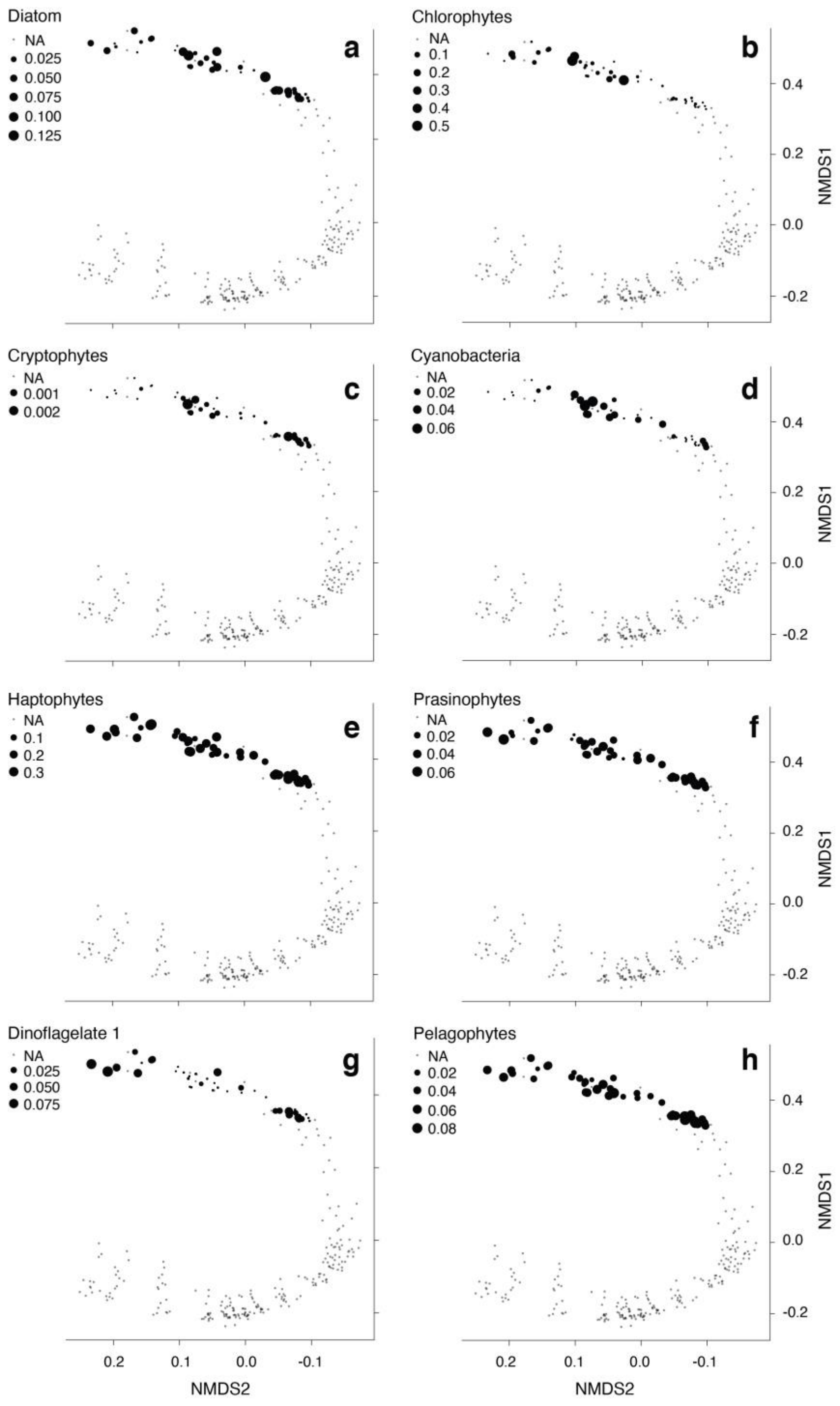

Figure S3. Bray-Curtis dissimilarity of all samples in a non-metric multidimensional scaling (nMDS) plot weighted by the pigment concentration of the eight different phytoplankton groups measured at OSP: (a) Diatoms, (b) Chlorophytes, (c) Cryptophytes, (d) Cyanobacteria, (e) Haptophytes, (f) Prasinophytes, (g) Dinoflagellates, and (h) Pelagophytes.

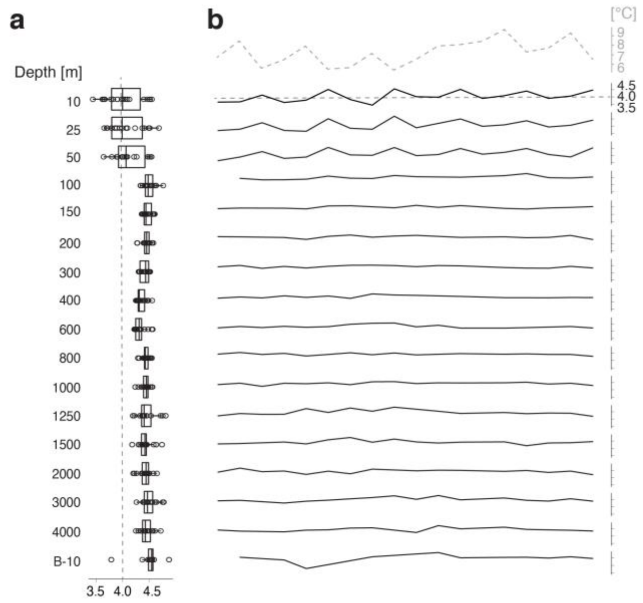

Figure S4. Alpha-diversity (Shannon Index) of the microbial communities at each depth shown as a) a boxplot (centre line represents the median, the lower box limit the 25% quartile, the upper box limit the 75% quartile, and the whiskers extend to 1.5 * IQR) and b) over time. The sparkline is the mean temperature, between 10 to 100 m depth, over time.

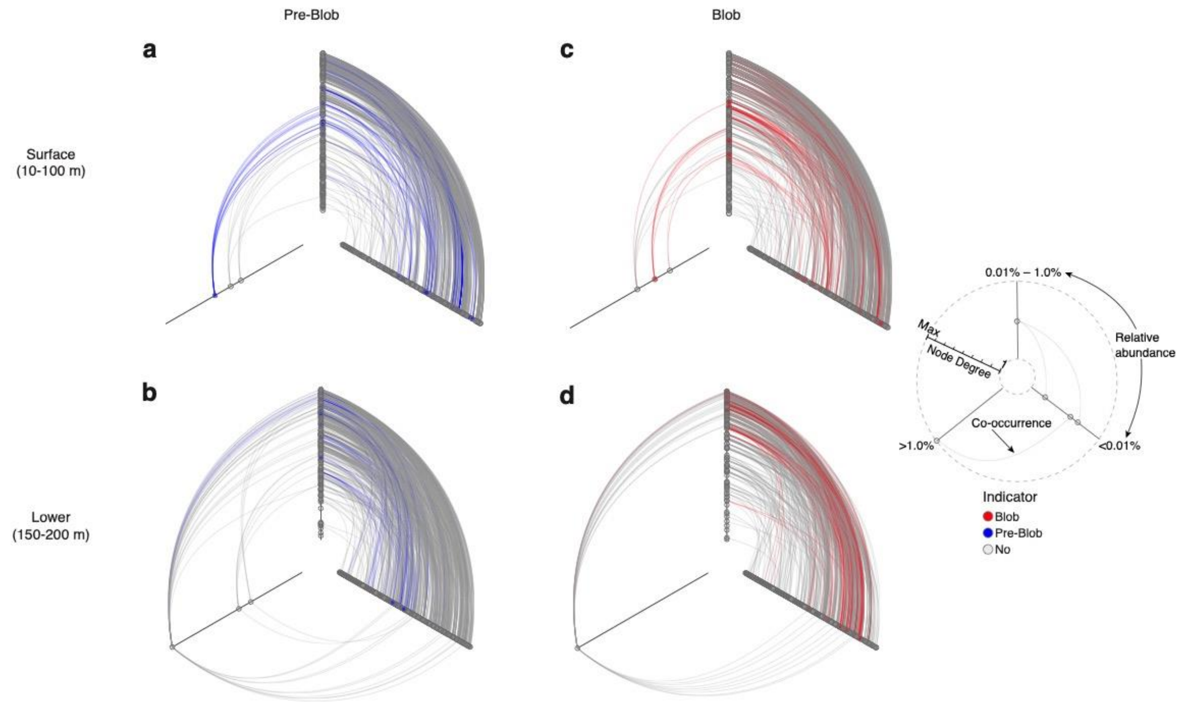

Figure S5. Hive plot panel of all positive edges from the network analyses of the communities in the 10-100 m (Surface) and 150-200 m (Lower) of the water column, in pre-Blob (a, c) and Blob (b, d) years. Each circle represents an OTU or node in a network. Each hive plot has 3 axes which represent relative abundance bins (<0.01%, 0.01%-1.0%, and >1%) and nodes are place on an axis according to its relative abundance in each network. The position of the node along the axis is based on their number of positive edges (i.e. their node degree) in the network. The maximum node degree varies by network: (a) 65, (b) 51, (c) 60, (d) 33. Node color denotes whether it was an indicator for pre-Blob conditions (blue) or Blob conditions (red) and met the threshold of 3 or more positive edges in the network. Grey circles represent nodes in the network that were either not indicators or were indicators but did not have at least three edges.

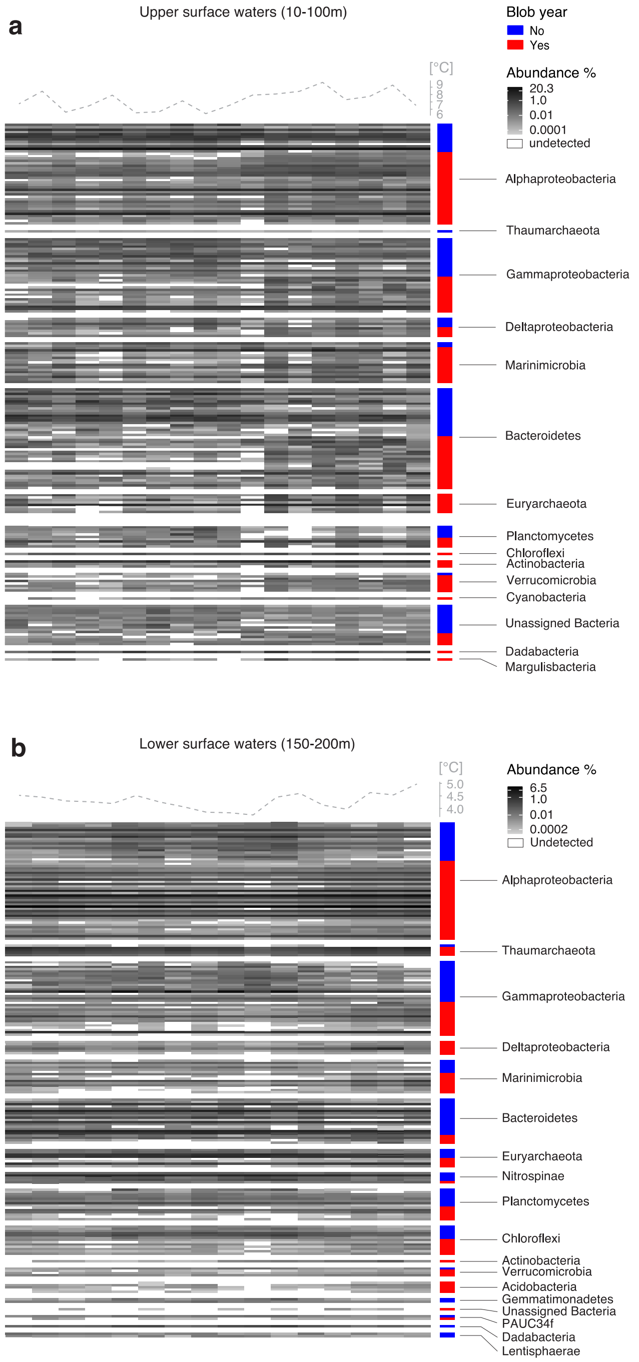

Figure S6. heatmap of relative abundance of indicator OTUs in a) the upper (10-100 m) and b) lower (150-200 m) part of the water column. Heatmap colors were rescaled using a log normalization, for better representing low abundant OTUs.

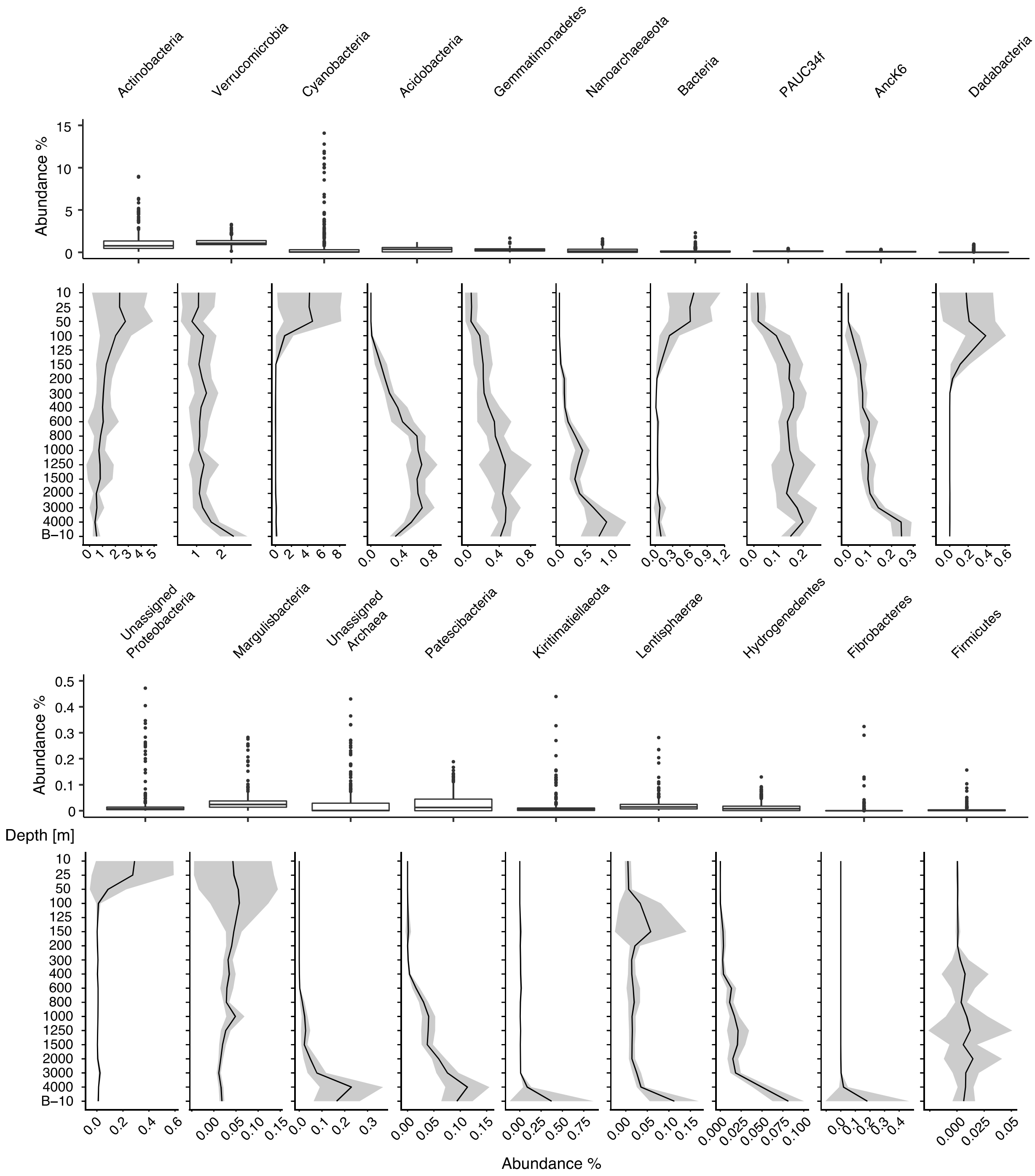

Figure S7. Remaining phyla identified in OSP communities not shown in Figure. 4, ranked by total relative abundance across the entire dataset shown in boxplots (centre line represents the median, the lower box limit the 25% quartile, the upper box limit the 75% quartile, and the whiskers extend to 1.5 * IQR), and their relative abundance and range in the water column.

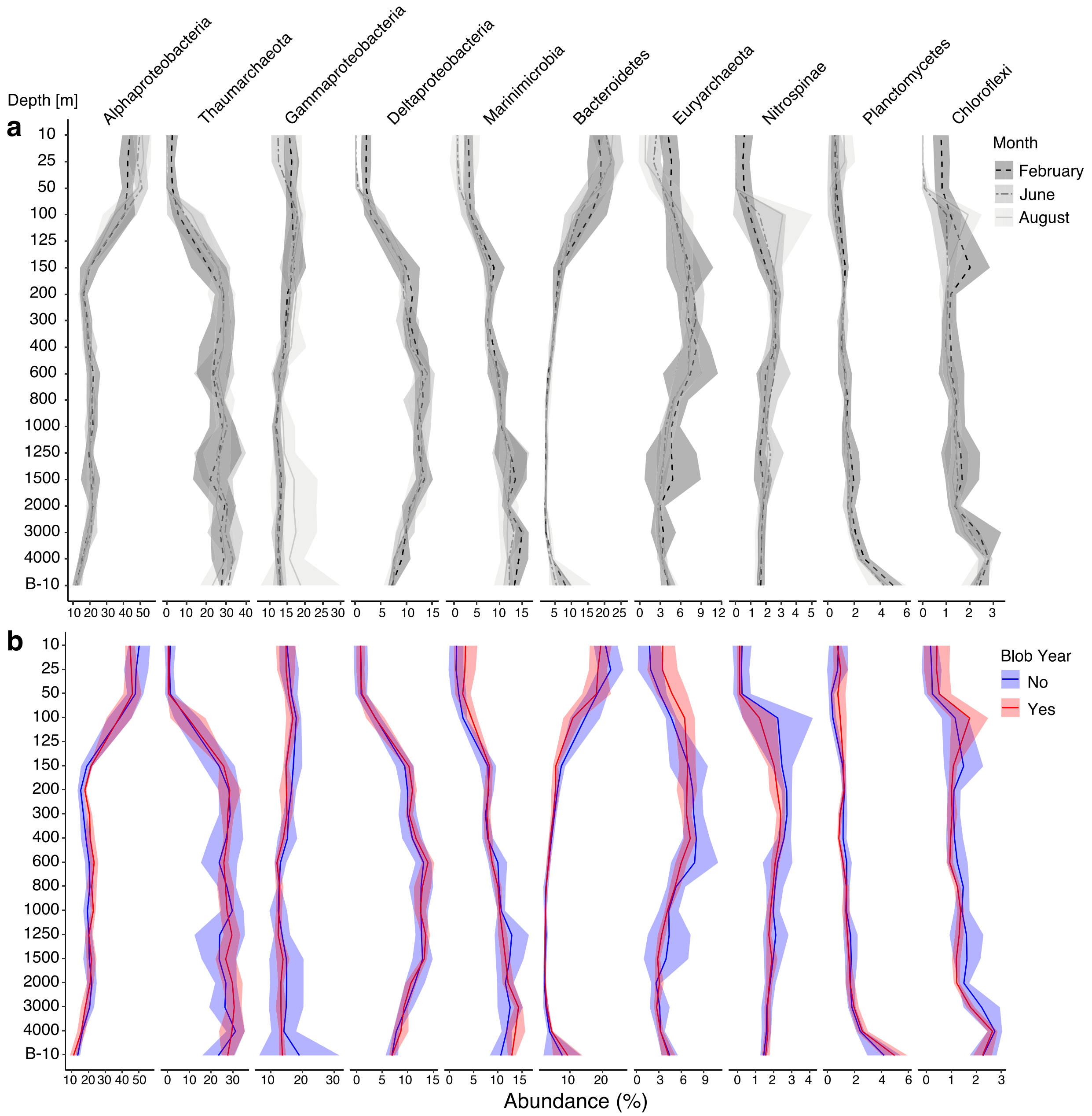

Figure S8. The average relative abundance (and range) distribution along the vertical depth profile for the 10 most abundant phyla ranked by total relative abundance across the entire dataset partitioned by a) sampling month and b) whether they came from a Blob (yes) or pre-Blob (no) year.

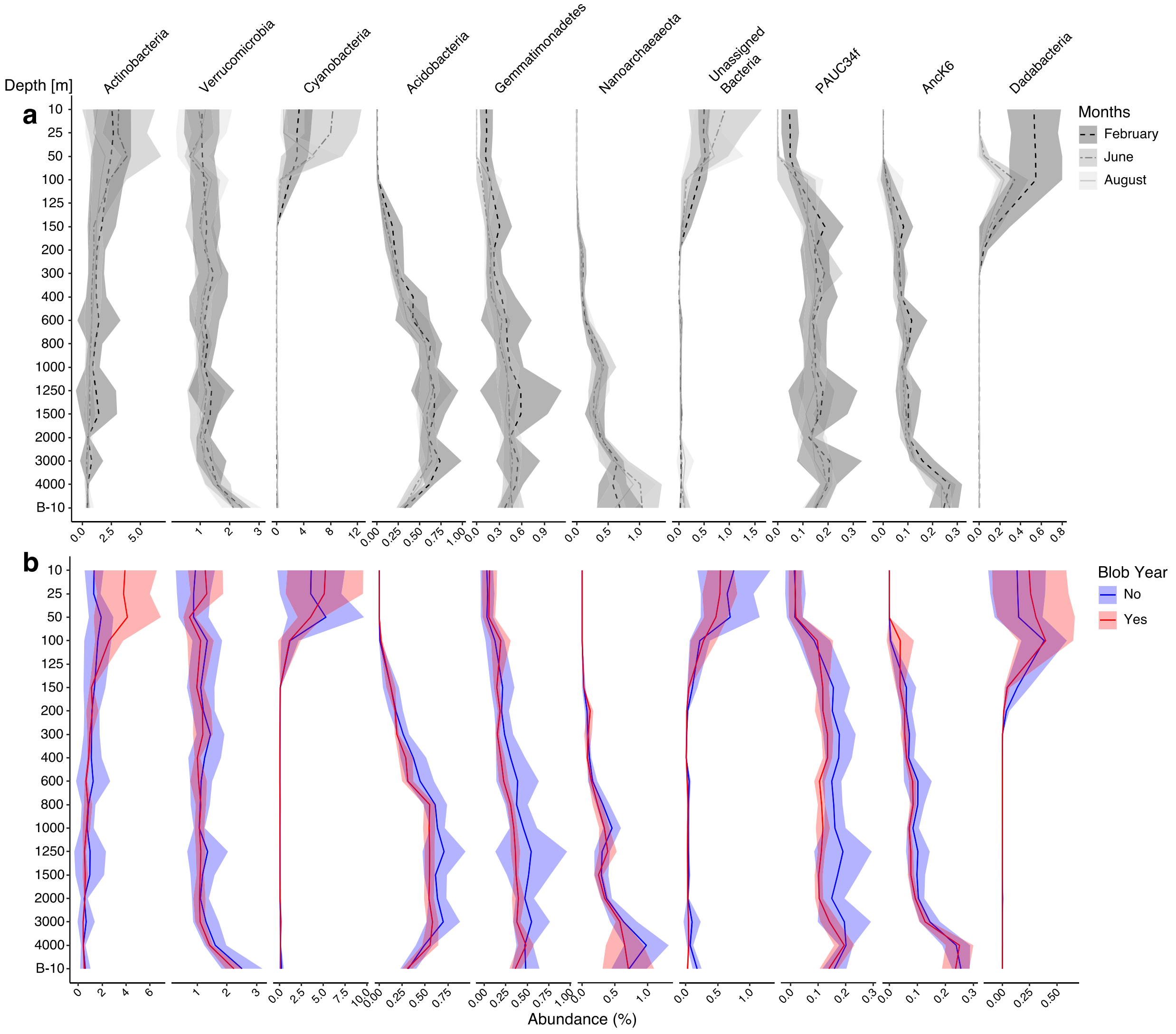

Figure S9. The average relative abundance (and range) distribution along the vertical depth profile for the 11-20 most abundant phyla ranked by total relative abundance across the entire dataset partitioned by a) sampling month and b) whether they came from a Blob (yes) or pre-Blob (no) year.

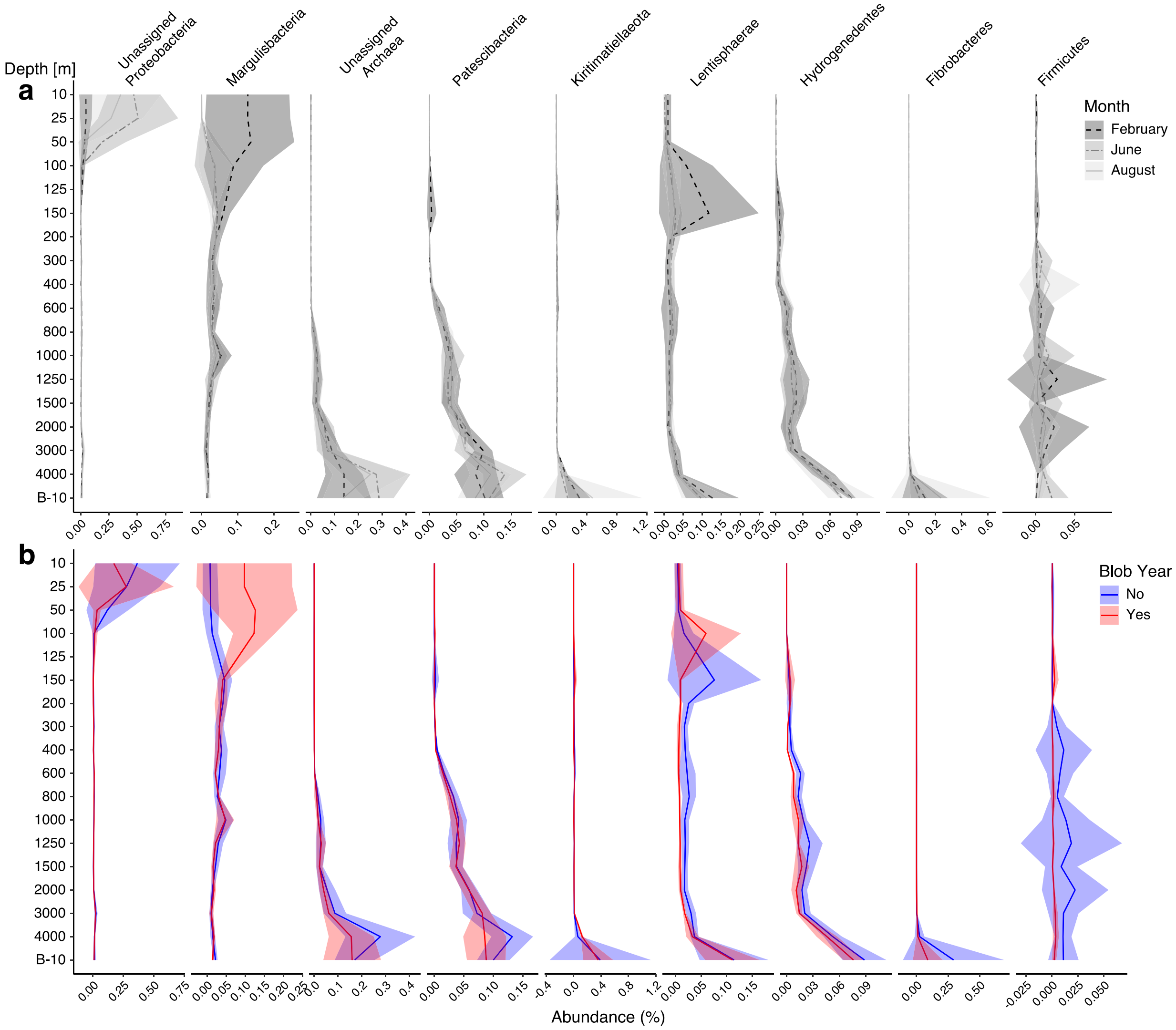

Figure S10. The average relative abundance (and range) distribution along the vertical depth profile for the 9 least abundant phyla ranked by total relative abundance across the entire dataset partitioned by a) sampling month and b) whether they came from a Blob (yes) or pre-Blob (no) year.

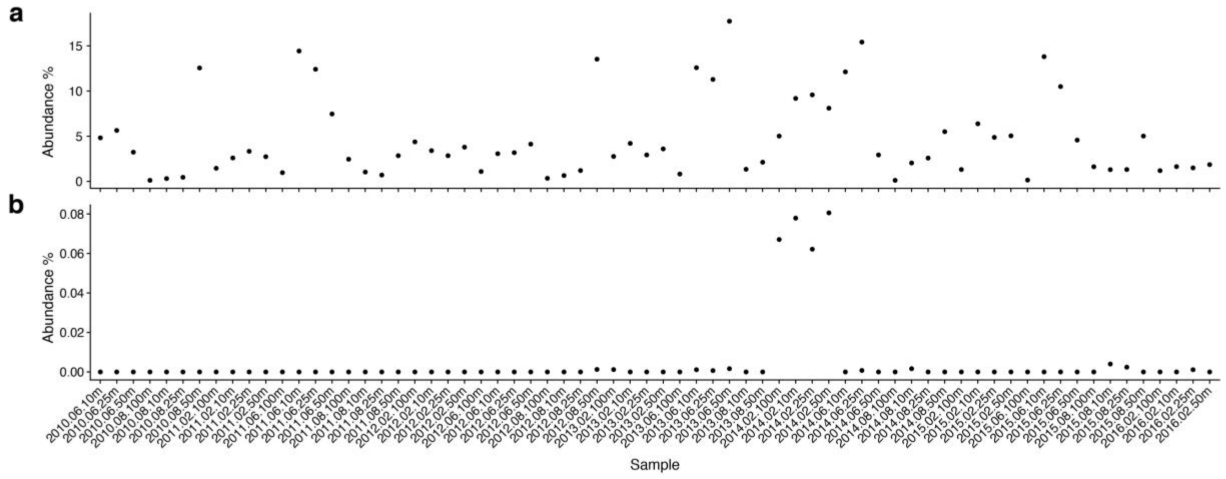

Figure S11. Total relative abundance (%) of all OTUs assigned at the genus level to a) *Synechococcus* and b) *Prochlorococcus*, in the OSP amplicon dataset. Please notice that sample order is chronological but not ordered by depth.

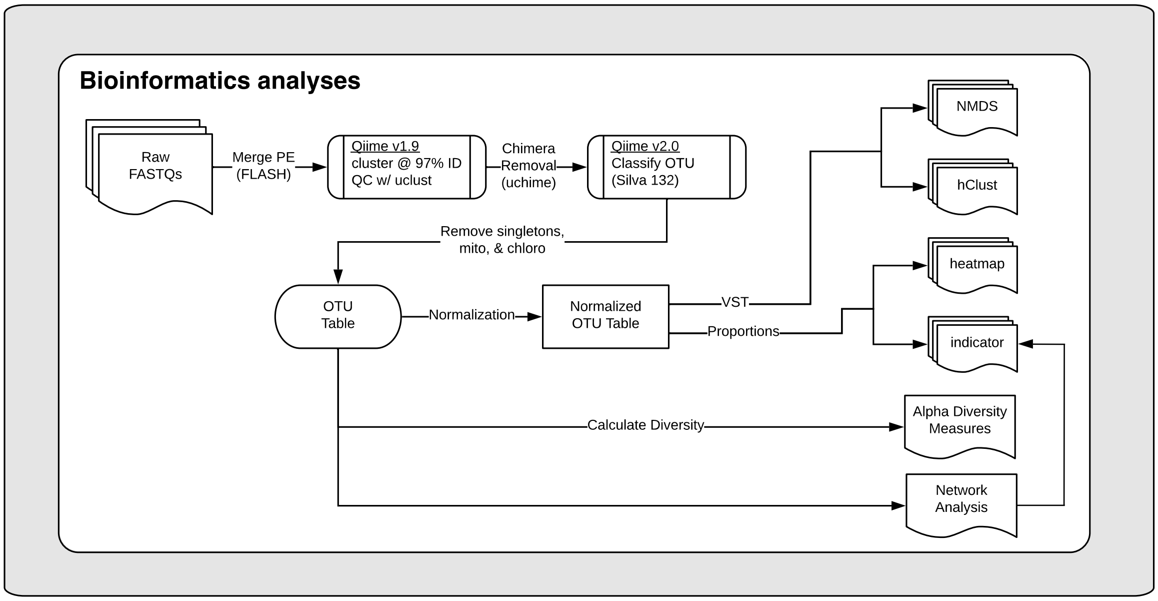

Figure S12. Bioinformatics processing of samples. The OTU table was generated from amplicons of the V4-V5 region of the 16S rRNA gene, clustered at 97% similarity. The OTU table were underwent quality control before being analyzed, see also Materials and Methods. All analyses of the OTU table were performed on either the raw abundances, normalized abundances using variance stabilization transformation (VST), or proportional abundances as indicated in the workflow. A RMarkdown (Blob_MS_figures_v32.Rmd) file with the relevant input files (Blob_markdown_input.zip) of all analyses and graphs produced in R, and the files required for running the network analysis (Blob_settings.config, Blobsubset_depths.cys and Blob_subset_depths.gephi) is provided as supplementary material. All source material can also be found here: https://github.com/hallamlab/Blob.

Supplementary Discussion

Ocean Station Papa Conceptual Model Description and Rationale

Background: The northeast subarctic Pacific (NESAP) Ocean experienced a marine heatwave (MHW), termed "The Blob" beginning in the winter of 2013-2014 and persisting through winter 2016. In addition to warmer surface ocean temperatures, increased stratification, reduced mixing, and lower inorganic nutrients were observed. Phytoplankton biomass experienced a 50% drop in the first year of the heatwave but then rebounded, with a different community characterized by smaller cells, including cyanobacteria[1,2]. Using small subunit ribosomal (SSU or 16S rRNA) gene amplicon sequence data sourced from Ocean Station Papa (OSP), the terminal station along the Line P transect in the NESAP, we observed that the prokaryotic community also experienced a change from taxa commonly associated with particles to those more associated with free-living lifestyles.

Motivation: Based on these observations we decided to graphically document, in a streamlined way, observed and hypothesized changes in the NESAP food web and carbon system in response to The Blob as a proxy for the impact of marine heatwaves on the northeast Pacific Ocean.

Overall Design: We utilized the structure of a co-occurrence network, with nodes representing carbon pools and edges representing flow of carbon between these pools (Figure 5 in main text). The model has two panels; the left representing Normal Conditions (pre-2014) and the right representing Heatwave Conditions (winter 2013/2014 – Winter 2016).

In the Normal conditions panel, we based carbon pool size on averages constructed from a literature review (discussed in more detail below) and thus the relative differences are based on data. One exception here is DOC, because the concentration dissolved organic carbon in the ocean is far greater than any other carbon pool.

The focus of this conceptual model is on changes in carbon pools and not necessarily carbon flow, especially because measurements of the biological rates are rare at OSP. Therefore, for the purpose of this conceptual model, edges or connections between carbon pools are kept equal in size (even though in reality they different) and we highlight predicted deviations (increases or decreases) from the "normal conditions" in the "heatwave conditions" side of the model.

Relative changes in carbon pools and flow during a heatwave are noted in the Heatwave Conditions panel. These deviations from normal conditions are based on available data or hypotheses based on our understanding of the ocean system at Ocean Station Papa.

Specific model elements: Here we describe how we constrained carbon pool size in the Normal Conditions panel and what available NESAP data or observations from other ocean systems lead us to suggest specific changes under MHW conditions.

Phytoplankton

Chlorophyll *a* concentration is a standard oceanographic measurement and used as proxy for phytoplankton biomass e.g. production. Average phytoplankton biomass was estimated by

compiling literature values, often given as mg m^{-3} or µg L^{-1} of Chlorophyll *a*, and converting these to carbon, where applicable (described below).

**Study**	**Season**	**Literature Value**	**Units**	**Carbon in photic zone*** (mg C m^{-2})
Semeniuk et al. 2009[3]	Summer	0.21	µg Chl *a* L^{-1}	840
Boyd et al. 1995[4]	Winter	20-28	mg Chl *a* m^{-3}	1600-2240
Boyd et al. 1995	Spring	19.7-31.7	mg Chl *a* m^{-3}	1576-2536
Harrison 2002[5]	Winter	15	µg C L^{-1}	1200
Harrison 2002	Spring	25	µg C L^{-1}	2000
Boyd and Harrison 1999[6]	Winter	0.2	µg Chl *a* L^{-1}	800
Boyd and Harrison 1999	Spring	0.4-0.6	µg Chl *a* L^{-1}	1600-2400
Boyd and Harrison 1999	Summer	0.2-0.4	µg Chl *a* L^{-1}	800-1600
**Average**				**1600**

*0-80 m

Conversion from µg Chl *a* L^{-1} to mg C m^{-3} using a carbon:chlorophyll ratio of 50 (as per Booth et al 1993).

$$(eq. 1) \qquad \frac{X\ \mu g\ Chl\ a}{L} * \frac{50\ \mu g\ C}{\mu g\ Chl\ a} * \frac{1\ L}{0.001\ m^3} = \frac{X\ \mu g\ C}{m^3}$$

To estimate the pool of phytoplankton-derived carbon in the photic zone, the concentration of carbon was multiplied by the depth of the photic zone. Although this depth was not always reported numerous studies conducted on OSP waters indicate that it ranges between 25 m and ~100 m [5,7–10]. While the depth of the photic zone and the depth of the mixed layer vary seasonally, a fixed value between 80 and 100 m is often employed for modelling purposes (for example Vézina and Savenkoff 1999[11]). **We selected to integrate our values over the 0-80 m because some of the values we were able to pull from the literature were only integrated over this depth range.**

Zooplankton

Ocean Station Papa (formerly Ocean Weather Station P) has a rich history of zooplankton research detailing community structure and biomass, especially between 1956 and 1980 as well as in the context of Joint Global Ocean Fluxes Study (JGOFS, http://ijgofs.whoi.edu/) and Global Ocean Ecosystem Dynamics (GLOBEC, http://www.igbp.net/) programs. Zooplankton biomass is estimated using net tows and often reported according to the number of individuals m^{-3} for taxa of interest. While biomass is often reported as wet weight or dry weight of dominant taxa or entire net tow, occasionally, the whole biomass of the net tow is reported in units of carbon (mg C m^{-3}). Some of the studies reported these data are summarized below.

**Study**	**Season**	**Literature Value**	**Units**	**Carbon in 150 m tow** (mg C m^{-2})	**Carbon in photic zone*** (mg C m^{-2})
Goldblatt et al. 1999[12]	Spring	20	mg C m^{-3}	3000	1600
Goldblatt et al. 1999	Winter	3	mg C m^{-3}	450	240
Parsons and Lalli 1988[7]	Winter	30	mg wet weight m^{-3}	360	192
Parsons and Lalli 1988	Spring	50-125	mg wet weight m^{-3}	600-1500	320-800
Marlowe and Miller 1975[13]	Winter	10-30	g wet weight 1000 m^{-3}	120-360	64-192
Marlowe and Miller 1975	Spring	50-175	g wet weight 1000 m^{-3}	600-2100	320-1120
**Average**				**970**	**517**

*0-80 m

Assuming zooplankton dry weight is on average between 10-30% of wet weight and carbon content is 20-50% of dry weight[14], we estimated carbon using the following equation:

$$(eq. 2) \qquad \frac{X\ mg\ wet\ weight}{m^3} * \frac{0.2\ g\ dry\ weight}{g\ wet\ weight} * \frac{0.4\ mg\ C}{g\ dry\ weight} = \frac{mg\ C}{m^3}$$

Zooplankton tows are often carried out over the upper 150 m at OSP and thus we can calculate the biomass per cubic meter and convert to square meter by multiplying by this depth. By integrating zooplankton biomass over depth, we can compare with phytoplankton biomass estimates, which we also integrated over the depth of the photic zone (80 m for this exercise).

Microzooplankton (heterotrophic protists)

It is important to note that the above estimates of zooplankton biomass do not include heterotrophic protists (sometimes referred to a microzooplankton in food web models). While the size of these organisms is small, they play a crucial role in grazing of both phytoplankton and bacteria and as a food source for zooplankton, especially in the fall and winter when their biomass has been shown to outweigh that of mesozooplankton in the western subarctic Pacific off the coast of Japan[15].

**Study**	**Season**	**Literature Value**	**Units**	**Carbon in photic zone*** (mg C m^{-2})
Booth et al. 1993[16]	Spring	890-990	mg C m^{-2}	(see lit value)
Booth et al. 1993	Late summer	760-1311	mg C m^{-2}	(see lit value)
Boyd et al. 1995[4]	Winter	7-13	mg C m^{-3}	560-1040
**Average**				**925**

*0-80 m

<u>Prokaryotes</u>

Prokarytic biomass is estimated from cell counts obtained using epifluorescence microscopy or, more recently, by flow cytometry. The number of cells is then multiplied by a carbon conversion factor. Many studies in the NESAP use a conversion factor of 20 fg C $cell^{-1}$ (Lee and Fuhrman 1987) although some studies suggest it could be as low as 12 fg C $cell^{-1}$ (Fukuda et al. 1998). For consistency with OSP literature we will use 20 fg C $cell^{-1}$, recognizing that it may be an overestimate.

**Study**	**Season**	**Literature Value**	**Units**	**Carbon in photic zone*** (mg C m^{-2})
Sherry et al. 1999[9]	Winter	9-13	µg C L^{-1}	720-1040
Sherry et al. 1999	Spring and Summer	21-27	µg C L^{-1}	1680-2160
Semeniuk et al 2009[3]	Late Summer	5.35	x10^8 cells L^{-1}	856
Kirchman 1993[8]	Spring and Summer	497-1764	mg C m^{-2}	(see lit value)
**Average**				**1245**

*0-80 m

$$(eq.\ 3)\qquad \frac{5.35\ x\ 10^{8}\ cells}{L} * \frac{20\ x\ 10^{-15} g\ C}{cell} * \frac{1\ L}{0.001\ m^3} * \frac{1000\ mg}{g} = \frac{10.7\ mg\ C}{m^3}$$

<u>Viruses</u>

To our knowledge, viral abundances have not yet been estimated from OSP. Therefore, we used virus to microbial cell abundance ratios (VMR) and the estimated biomass of a virus to convert from bacterial biomass to viral biomass. A recent re-examination of VMR showed that the median virus to microbial cell ratio from samples <100 m was 11.13[17]. The range of carbon content of viruses is quite large, from 0.055 up to 0.2 fg C per virus [18,19] and modeled to be as low as 0.02 fg C per virus[20]. We used these values to create a conversion factor that does not require cell counts and can leverage the bacterial biomass values reported in some OSP papers.

$$(eq. 4 - \text{MIN}) \quad viral\ biomass = \frac{0.055\ fg\ C}{virus} * \frac{11\ virus}{bacteria} * \frac{bacteria}{20\ fg\ C} * average\ bacterial\ biomass$$

$$(eq. 5 - \text{MAX}) \quad viral\ biomass = \frac{0.2\ fg\ C}{virus} * \frac{11\ virus}{bacteria} * \frac{bacteria}{20\ fg\ C} * average\ bacterial\ biomass$$

Therefore, with an average OSP bacterial biomass of 1245 mg C m^{-2}, it follows that viral biomass ranges from **37.7** to **137 mg C m^{-2}**. It is important to note that the VMR is an underestimate given the prevalence of RNA viruses in the ocean and the fact that current direct count estimates of viruses may miss many of these viruses due to the nucleic acid stain used and the small size of many RNA viruses[21] (personal communication with C. Suttle). We can attempt to account for these as follows: (1) If we assume that the ratio of viruses to protists is the same as for viruses to prokaryotes (even though it is possible that this ratio is even higher, given large burst sizes of eukaryotic viruses[21]), and (2) that an RNA virus, many of which are suspected to be very small, has a biomass of 0.02-0.05 fg C $virus^{-1}$. The surface (2-25 m) concentration of protists reported by Booth et al. 1993 was $2.7x10^{7}$ autotrophic cells and $1.5x10^{6}$ heterotrophic cells. If we triple this number to approximate the number of protists between 0 and 80 m, then the approximate number of RNA viruses in the photic zone would be 9.34 $x10^{8}$ and at 0.05 fg C $virus^{-1}$, the biomass of RNA viruses would be 0.05 mg C m^{-2}. But, work by Steward et al. 2013 suggest that the number of RNA viruses in the ocean may be equivalent to that of dsDNA viruses (e.g. viruses counted by current microscopic or flow cytometric methods). In this case, the biomass of viruses in the ocean would be double of the estimates given above. So, perhaps instead of 37.7 mg C m^{-2} **viral biomass could be as much as 78 mg C m^{-2}** (or even higher, depending on the estimate of carbon per viruses used).

<u>DOC</u>

Dissolved organic carbon (DOC) represents the largest biologically active pool of carbon in the ocean, nearly equivalent to the amount of atmospheric CO_2[22]. The concentration of DOC at OSP is often quantified in the context of better estimating net community production (NCP). Traditionally measured using via the collection of GF-F filtered seawater and analysis on a Shimadzu TOC system, DOC is also able to be estimated using Bio-Argo floats (which measure temperature, Salinity, and nitrate).

DOC is often reported in units of μmol C kg^{-1} and thus a conversion to mg C m^{-2} is necessary. A seawater density of 1.025 kg L^{-1} is employed for this conversion.

Study	Season	Literature Value	Units	Carbon in photic zone* (mg C m^{-2})
Bif and Hansell 2019 [22]	Winter	56	μmol kg^{-1}	55104
Bif and Hansell 2019	Summer	73	μmol kg^{-1}	71832
Wong et al. 2002[23]	Winter	65	μmol kg^{-1}	63960
Wong et al. 2002	Spring	80	μmol kg^{-1}	78720
**Average**				**67404**

*0-80 m

$$(eq. 6) \qquad \frac{[DOC]\ \mu mol\ C}{kg} * \frac{1.025\ kg}{L} * \frac{1000\ L}{m^3} = \frac{[DOC]\ \mu mol\ C}{m^3} * \frac{1\ mol}{1E6\ \mu mol} * \frac{12\ g}{mol} = \frac{[DOC]\ g}{m^3}$$

DOC concentration can then be converted to mg m^{-3} and the multiplied by the depth of the photic zone (using 80 m for this exercise).

POC

Estimates of particulate organic carbon (POC) concentrations are fundamental to understanding the marine carbon cycle and, in the open ocean where allochthonous inputs of particulates, often relate to how productive the ecosystem is. POC concentrations at OSP were measured as part of the Canadian JGOFS).

Study	Season	Literature Value	Units	Carbon in photic zone (mg C m^{-2})
Bishop et al. 1999[24]*	Winter	133-235	mmol C m^{-2}	1596-2820
Bishop et al. 1999	Spring	175-191	mmol C m^{-2}	2100-2292
Bishop et al. 1999	Summer	222.5-227.6	mmol C m^{-2}	2670-2731
**Average**				**2368**

* values integrated over 0-100 m in this Bishop et al.

$$\frac{[POC]\ mM\ C}{m^2} * \frac{1\ mol}{1000\ mmol} * \frac{12\ g\ C}{mol} * \frac{1000\ mg}{g} = mg\ C\ m^{-2}$$

CO_2 flux IN and OUT

Air-sea fluxes of CO_2 have been estimated using sensors deployed on moorings at OSP and bio-Argo floats present in the region, often as means to estimate net community production[10]. These studies estimate the OSP is net autotrophic in the spring and summer (March-September) and then

switches to net heterotrophic from October to February. The carbon demand in the spring and summer is as much as **3 mol C m^{-2}** (36 g C m^{-2}; IN arrow) and closer to **1 mol C m^{-2}** (12 g C m^{-2}) in the winter (OUT arrow), with net community production close to 2 mol C m^{-2}. If we want to estimate the daily flux, then we can divide the flux in by 210 days (7 months) to get and the flux out by 150 days (5 months).

<u>POC Flux</u>

At 50 m, POC flux is estimated at 11.3 mmol C m^{-2} d^{-1} or 135.6 mg C m^{-2} d^{-1}. At 100 m, POC flux is estimated at 6.1 mmol C m^{-2} d^{-1} or **73.2 mg C m^{-2} d^{-1}** [23]. At 200 m, winter POC flux values around 6 mg C m^{-2} d^{-1} and spring values up to 38 mg C m^{-2} d^{-1} and an annual flux of 6.63 g C m^{-2} yr^{-1} [25]. We will use the 100 m values for this conceptual model.

Predicted changes during a marine heatwave

<u>Phytoplankton:</u> To assess the potential changes in phytoplankton biomass in response to marine heatwaves, we gathered chlorophyll concentrations (from Angelica Peña, DFO) from the Line P cruises that we have prokaryotic community data from. We then averaged the photic zone concentrations (0-50 m) during pre-Blob conditions ('Normal conditions', 2010-Aug 2013) and during the Blob ('Heatwave conditions', 2014- Feb 2016), to estimate the % change in phytoplankton biomass. Average chlorophyll concentration during Normal conditions was 0.405 ± 0.109 and 0.434 ± 0.145 during Heatwave conditions. By averaging in this manner, we see that overall there was a trend that phytoplankton biomass **increased by ~11%** during The Blob, but it is important to note that during the first year of the temperature anomaly, biomass actually decreased to 60% of normal, and then rebounded from Aug 2015 onwards. Thus, the response of the phytoplankton community to MHWs is expected to be dynamic and not the same throughout the duration of the heatwave. During much of the Blob, phytoplankton community composition changed to be dominated by smaller cells including cyanobacteria and chlorophytes. Diatom pigment concentrations were ~40% lower than normal, even considering their recovery by the end of the Blob. Thus, we change the ratio of big:small phytoplankton in the model.

<u>Zooplankton:</u> To assess the potential changes in zooplankton biomass in response to marine heatwaves, we gathered estimated zooplankton biomass data from 0-150 m bongo net tows (from Moira Galbraith, DFO) from the Line P cruises that we have prokaryotic community data from. During normal conditions average biomass was 33.9 ± 17.5 mg dry weight m^{-3} and 37.4 ± 15.7 mg dry weight m^{-3} during The Blob. Therefore, during the Blob, zooplankton biomass **increased by ~ 10%**. Even with this increase in biomass, there was a shift towards smaller zooplankton often associated with warmer waters off California and Oregon (e.g. positive anomalies in Calanus pacificus[26]). Thus, we change the ratio of big:small zooplankton in the model.

<u>Microheterotrophs:</u> Given the prevalence of smaller phytoplankton during the Blob and a likely increase in prokaryotes (more on this below), we predict a more robust microbial loop including an **increase in heterotrophic protists**. Rivkin et al. found that heterotrophic protists at OSP obtained most of their nutrition from prokaryotes and autotrophic picoplankton and were not as reliant on herbivory (of larger phytoplankton) and that their ingestion rates increased with warmer temperatures[27].

Prokaryotes: In the English channel, it was observed that warming ocean waters brought about an increase in bacterial production[28]. At OSP, Rivkin et al.[27] observed bacterial abundances to be significantly positively correlated with mixed layer temperature and Kirchman et al.[8] showed that increasing the temperature of their bacterial production assays resulted in immediately higher rates of substrate incorporation. Furthermore, temperature increases allowed the OSP bacteria to utilize DOM more efficiently. Given these observations, we predict that **prokaryotic biomass at OSP increases in response to marine heatwaves** and so too does DOM utilization. Also, with the shift from large to small phytoplankton assemblages, we observed a change in the prokaryotic community from typically particle-associated to free-living taxa.

Interestingly, in the western English Channel, it was observed that increasing temperature did not have the same effect on bacterial production across all seasons – warming temperatures brought about more substantial increases in winter bacterial production that in summer. Joint and Smale[28] suggested that summer communities were already operating at or near their optimal temperatures and thus an increase in water temperature would not bring about as significant of a response in production compared to winter communities, which were not operating near their optimal temperatures. Therefore, it is important to keep in mind that increasing ocean temperatures during MHWs may not always yield a positive prokaryotic response, especially when heatwave temperatures approach or exceed the optimal temperature range for indigenous microbial assemblages.

Viruses: Given the predicted increase in prokaryotic (and phytoplankton and zooplankton) biomass, we **anticipate the viral biomass will also likely increase** in response to marine heatwaves. This may contribute to an increase in the DOC pool, as more cells may be lysed during viral attack. This would be an excellent experimental study for future Line P cruises.

DOC: DOC concentrations derived from Bio-Argo profiles between Jan 2009 and March 2018 show an increase between 2014, accompanied by high net community production, followed by a decline between 2015, where very little observable net community production was measured in the spring and summer[22,29]. Thus, it is difficult to state that DOC increases or decreases in response the MHWs in a binary manner so we will keep [DOC] concentration the same. Bif et al. suggest that there was a resilience to the physical changes brought on by the Blob during 2014 due to the availability of recycled iron (and possible mismatch with grazers) but that this resilience was lost in 2015 with the availability of iron continued to decline due to stratification and reduced mixing[29].

POC and POC Flux: Given the overall increase in Chl *a* at OSP during the Blob, we **hypothesize that POC also increased.** This is consistent with satellite-derived estimates of Chl *a* and POC for the same time period[30]. However, given the very low net community production in 2015[29], the size shift in the phytoplankton-derived carbon throughout much of the heatwave[1,2], and the general shift to smaller zooplankton (and thus smaller fecal pellets), we suggest that more of this carbon was retained and processed within the surface ocean since small cells sink more slowly than larger cells (and thus a **decrease in POC flux**). The differences observed in phytoplankton community composition[1,2] undoubtedly influenced POC quality (not just quantity), which we propose had an important effect on prokaryotic community structure.

Net community production: Net community production estimates at OSP have ranged between ~1

and 2 mol C m^{-2} across many studies. Bif and Hansell[22] estimate that spring and summer NCP in 2014 was around 1.6 and 2.2 mol C m^{-2} respectively but that spring NCP was -0.3 mol C m^{-2} in the spring of 2015 and only 0.3 mol C m^{-2} in the summer of that year. Given that we know that phytoplankton biomass was actually higher in 2015 than in 2014, this suggests that the microbial loop was very active in this year and **heterotrophy was likely greater than normal.** Thus, we expect the flux OUT increased during the heatwave while the flux IN decreased (driven by to the large decline in NCP in 2015).

Linkages

Phytoplankton → Zooplankton – Decrease
Phytoplankton → Heterotrophic protists – Increase
Heterotrophic protists ← Bacteria – Increase
Phytoplankton → Bacteria – Decrease; since we observe a decline in particle-associated taxa and an increase in free-living taxa
Zooplankton ← Heterotrophic protists – Increase
Viruses ← Bacteria – Increase
Viruses ← Heterotrophic protists – Increase
Viruses ← Phytoplankton – Increase
Viruses ← Zooplankton – Increase
Viruses → DOC – Increase
Phytoplankton → DOC – Increase
Zooplankton → DOC – Increase
Bacteria ← → DOC – Increase
Heterotrophic protists → DOC – Increase
Phytoplankton → POC – Increase; since biomass increases, but the size will be more nano-sized detritus vs micro-sized detritus.
Zooplankton → POC – Decrease; since fecal pellet size decreases with smaller zooplankton
Bacteria ← POC – Decrease; since we observe a decline in particle-associated taxa and an increase in free-living taxa.
Heterotrophic protists ← POC – Increase
DOC ← POC – Increase

Supplementary References

1. Yang, B., Emerson, S. R. & Peña, M. A. The effect of the 2013-2016 high temperature anomaly in the subarctic Northeast Pacific (the 'Blob') on net community production. *Biogeosciences* **15**, 6747–6759 (2018).
2. Peña, M. A., Nemcek, N. & Robert, M. Phytoplankton responses to the 2014–2016 warming anomaly in the northeast subarctic Pacific Ocean. *Limnol. Oceanogr.* **64**, 515–525 (2019).
3. Semeniuk, D. M. *et al.* Plankton copper requirements and uptake in the subarctic Northeast Pacific Ocean. *Deep. Res. Part I Oceanogr. Res. Pap.* **56**, 1130–1142 (2009).
4. Boyd, P. *et al.* The NE subarctic Pacific in winter: I. Biological standing stocks. *Mar. Ecol. Prog. Ser.* **128**, 11–24 (1995).
5. Harrison, P. J. Station Papa time series: Insights into ecosystem dynamics. *J. Oceanogr.* **58**, 259–264 (2002).
6. Boyd, P. & Harrison, P. J. Phytoplankton dynamics in the NE subarctic Pacific. *Deep. Res. Part II Top. Stud. Oceanogr.* **46**, 2405–2432 (1999).
7. Parsons, T. R. & Lalli, C. M. Comparative oceanic ecology of the plankton communities of the subarctic Atlantic and Pacific oceans. in *Oceanogr. Mar. Biol. Annu. Rev.* (eds. Barnes, H. & Margaret, B.) 317–359 (Aberdeen University Press, 1988).
8. Kirchman, D. L., Keil, R. G., Simon, M. & Welschmeyer, N. A. Biomass and production of heterotrophic bacterioplankton in the oceanic subarctic Pacific. *Deep. Res. Part I* **40**, 967–988 (1993).
9. Sherry, N. D., Boyd, P. W., Sugimoto, K. & Harrison, P. J. Seasonal and spatial patterns of heterotrophic bacterial production, respiration, and biomass in the subarctic NE Pacific. *Deep. Res. Part II Top. Stud. Oceanogr.* **46**, 2557–2578 (1999).
10. Fassbender, A. J., Sabine, C. L. & Cronin, M. F. Net community production and calcification from 7 years of NOAA Station Papa Mooring measurements. *Global Biogeochem. Cycles* **30**, 250–267 (2016).
11. Vézina, A. F. & Savenkoff, C. Inverse modeling of carbon and nitrogen flows in the pelagic food web of the northeast subarctic Pacific. *Deep. Res. Part II Top. Stud. Oceanogr.* **46**, 2909–2939 (1999).
12. Goldblatt, R. H., Mackas, D. L. & Lewis, A. G. Mesozooplankton community characteristics in the NE subarctic Pacific. *Deep. Res. Part II Top. Stud. Oceanogr.* **46**, 2619–2644 (1999).
13. Marlowe, C. J. & Miller, C. B. Patterns of vertical distribution and migration of zooplankton at Ocean Station "P"1. *Limnol. Oceanogr.* **20**, 824–844 (1975).
14. Parsons, T. R., Takahashi, M. & Hargrave, B. Chemical Composition. in *Biological Oceanographic Processes* 37–60 (Pergamon, 1984). doi:https://doi.org/10.1016/B978-0-08-030765-7.50007-6
15. Shinada, A., Ikeda, T., Ban, S. & Tsuda, A. Seasonal dynamics of planktonic food chain in the Oyashio region, western subarctic Pacific. *J. Plankton Res.* **23**, 1237–1247 (2001).
16. Booth, B. C., Lewin, J. & Postel, J. R. Temporal variation in the structure of autotrophic and heterotrophic communities in the subarctic Pacific. *Progress in Oceanography* **32**, 57–99 (1993).
17. Wigington, C. H. *et al.* Re-examination of the relationship between marine virus and microbial cell abundances. *Nat. Microbiol. 2016 13* **1**, 1–9 (2016).
18. Steward, G. F., Montiel, J. L. & Azam, F. Genome size distributions indicate variability and

similarities among marine viral assemblages from diverse environments. *Limnol. Oceanogr.* **45**, 1697–1706 (2000).

19. Wilhelm, S. W. & Suttle, C. A. Viruses and Nutrient Cycles in the Sea. *Bioscience* **49**, 781–788 (1999).
20. Jover, L. F., Effler, T. C., Buchan, A., Wilhelm, S. W. & Weitz, J. S. The elemental composition of virus particles: Implications for marine biogeochemical cycles. *Nat. Rev. Microbiol.* **12**, 519–528 (2014).
21. Steward, G. F. *et al.* Are we missing half of the viruses in the ocean? *ISME J.* **7**, 672–679 (2013).
22. Bif, M. B. & Hansell, D. A. Seasonality of Dissolved Organic Carbon in the Upper Northeast Pacific Ocean. *Global Biogeochem. Cycles* **33**, 2018GB006152 (2019).
23. Wong, C. S., Waser, N. A. D., Whitney, F. A., Johnson, W. K. & Page, J. S. Time-series study of the biogeochemistry of the North East subarctic Pacific: Reconciliation of the Corg/N remineralization and uptake ratios with the Redfield ratios. *Deep. Res. Part II Top. Stud. Oceanogr.* **49**, 5717–5738 (2002).
24. K.B. Bishop, J., E. Calvert, S. & Soon, M. Y. S. Spatial and temporal variability of POC in the northeast subarctic Pacific. *Deep. Res. Part II Top. Stud. Oceanogr.* **46**, 2699–2733 (1999).
25. Wong, C. S. *et al.* Seasonal and interannual variability in particle fluxes of carbon, nitrogen and silicon from time series of sediment traps at Ocean Station P, 1982-1993: Relationship to changes in subarctic primary productivity. *Deep. Res. Part II Top. Stud. Oceanogr.* **46**, 2735–2760 (1999).
26. Fisher, J. *et al.* Copepod responses to, and recovery from, the recent marine heatwave in the Northeast Pacific. in *PICES Press* **28**, 65–71 (2020).
27. Rivkin, R. B., Putland, J. N., Robin Anderson, M. & Deibel, D. Microzooplankton bacterivory and herbivory in the NE subarctic Pacific. *Deep. Res. Part II Top. Stud. Oceanogr.* **46**, 2579–2618 (1999).
28. Joint, I. & Smale, D. A. Marine heatwaves and optimal temperatures for microbial assemblage activity. *FEMS Microbiol. Ecol.* **93**, 243 (2017).
29. Bif, M. B., Siqueira, L. & Hansell, D. A. Warm Events Induce Loss of Resilience in Organic Carbon Production in the Northeast Pacific Ocean. *Global Biogeochem. Cycles* **33**, 1174–1186 (2019).
30. Yu, J., Wang, X., Fan, H. & Zhang, R. H. Impacts of Physical and Biological Processes on Spatial and Temporal Variability of Particulate Organic Carbon in the North Pacific Ocean during 2003–2017. *Sci. Rep.* **9**, 1–15 (2019).
