# Supplementary material for: Prokaryotic responses to a warm temperature anomaly in northeast subarctic Pacific waters
Source: Commun Biol. 2021 Oct 22;4:1217. doi: 10.1038/s42003-021-02731-9 (PMC8536700; doi:10.1038/s42003-021-02731-9)
Supplement: Supplementary file 3 — Description of Additional Supplementary Files [file 42003_2021_2731_MOESM3_ESM.pdf]

Description of Additional Supplementary Files

File name: Supplementary Data 1

Description: Sequence information for all samples. Columns B-E contain accession information to the National Centre for Biotechnology Information (NCBI) Short Read Archive (SRA) data repository. Columns H-Y contain information on read processing and QC, provided by the DOE Joint Genome Institute (JGI).

File name: Supplementary Data 2

Description: Environmental parameters and categorical definitions needed for the analysis of the amplicon data.
