# Supplementary material for: Prokaryotic responses to a warm temperature anomaly in northeast subarctic Pacific waters
Source: Commun Biol. 2021 Oct 22;4:1217. doi: 10.1038/s42003-021-02731-9 (PMC8536700; doi:10.1038/s42003-021-02731-9)
Supplement: Supplementary file 6 — Reporting summary [file 42003_2021_2731_MOESM6_ESM.pdf]

nature research

Corresponding author(s): Steven J. Hallam

Last updated by author(s): 11-02-2021

Reporting Summary

Nature Research wishes to improve the reproducibility of the work that we publish. This form provides structure for consistency and transparency in reporting. For further information on Nature Research policies, see our Editorial Policies and the Editorial Policy Checklist.

Statistics

For all statistical analyses, confirm that the following items are present in the figure legend, table legend, main text, or Methods section.

n/a	Confirmed	
☐	☒	The exact sample size (*n*) for each experimental group/condition, given as a discrete number and unit of measurement
☐	☒	A statement on whether measurements were taken from distinct samples or whether the same sample was measured repeatedly
☐	☒	The statistical test(s) used AND whether they are one- or two-sided<br>*Only common tests should be described solely by name; describe more complex techniques in the Methods section.*
☐	☒	A description of all covariates tested
☐	☒	A description of any assumptions or corrections, such as tests of normality and adjustment for multiple comparisons
☐	☒	A full description of the statistical parameters including central tendency (e.g. means) or other basic estimates (e.g. regression coefficient) AND variation (e.g. standard deviation) or associated estimates of uncertainty (e.g. confidence intervals)
☐	☒	For null hypothesis testing, the test statistic (e.g. *F*, *t*, *r*) with confidence intervals, effect sizes, degrees of freedom and *P* value noted<br>*Give P values as exact values whenever suitable.*
☒	☐	For Bayesian analysis, information on the choice of priors and Markov chain Monte Carlo settings
☒	☐	For hierarchical and complex designs, identification of the appropriate level for tests and full reporting of outcomes
☒	☐	Estimates of effect sizes (e.g. Cohen's *d*, Pearson's *r*), indicating how they were calculated

Our web collection on statistics for biologists contains articles on many of the points above.

Software and code

Policy information about availability of computer code

Data collection	The sequence data was generated on an Illumina Miseq using Illumina proprietary software.
Data analysis	All code and files for data analyses are provided as supplementary files in the submission and are also available at https://github.com/hallamlab/BLOB. The network analyses were performed using the Conet plugin for Cytoscape, Conet version 1.1.1 and visualization of networks was done using Gephi version 0.9.2 and Hive Panel Explorer (Sarah E I Perez, Aria S Hahn, Martin Krzywinski, Steven J Hallam, Hive Panel Explorer: an interactive network visualization tool, Bioinformatics, 2020;, btaa683, https://doi.org/10.1093/bioinformatics/btaa683). All required config and input files are supplied in the submission. all other analyses and graphs are produced using R version 3.4.4 and RStudio version 1.1.447, all packages used are provided in a Rmarkdown file as supplementary material.

For manuscripts utilizing custom algorithms or software that are central to the research but not yet described in published literature, software must be made available to editors and reviewers. We strongly encourage code deposition in a community repository (e.g. GitHub). See the Nature Research guidelines for submitting code & software for further information.

Data

Policy information about availability of data

All manuscripts must include a data availability statement. This statement should provide the following information, where applicable:

- Accession codes, unique identifiers, or web links for publicly available datasets
- A list of figures that have associated raw data
- A description of any restrictions on data availability

The DNA sequences of the 16S rRNA gene amplicons are available from the NCBI Sequence Read Archive under the BioProject no. PRJNA640752, accession no. SRR12059679-SRR12059949 https://trace.ncbi.nlm.nih.gov/Traces/sra_sub/sub.cgi?acc=SRP268127&focus=SRP268127&from=list&action=show:STUDY (the sequence data is currently not publicly available but will be released upon publication. It can be provided upon request to the corresponding author). All

environmental chemical parameters are presented in full in table 3, with the exception for the temperature data used for figure 1, this is freely available from www.waterproperties.ca

Field-specific reporting

Please select the one below that is the best fit for your research. If you are not sure, read the appropriate sections before making your selection.

☐ Life sciences ☐ Behavioural & social sciences ☒ Ecological, evolutionary & environmental sciences

For a reference copy of the document with all sections, see nature.com/documents/nr-reporting-summary-flat.pdf

Ecological, evolutionary & environmental sciences study design

All studies must disclose on these points even when the disclosure is negative.

Study description	The presented dataset consists of a total of 271 biological samples collected at Ocean Station Papa (P26, 50 °N, 145 °W) from CCGS John P. Tully during Line P cruises between June 2010 and February 2016.
Research sample	2 L seawater samples filtered onto 0.2 micrometer sterivex filters.
Sampling strategy	The biological samples were collected at fixed depths as part of a pre-established oceanographic time series. 2 L seawater samples was collected from niskin bottles attached on a rosette.
Data collection	Seawater was filtered onto a 0.2 micrometer sterivex filter, preserved in Sucrose Lysis Buffer and stored at -70 celsius degrees until DNA extraction. Samples were collected by authors and past members of the Hallam Lab, University of British Columbia, Canada. Sequence data was genered by the DOE Joint Genome Institute, Lawrence Berkeley National Laboratory, CA, USA. Environmental chemical data was collected by the Institute of Ocean Sciences (IOS), Sidney, Canada.
Timing and spatial scale	Sampling period June 2010 to February 2016, with three cruises per year. The Line P cruises run three times a year usually in the months February, June, and August. Samples were collected from station P26, the end station of the Line P transect, spanning 10 m below the surface down to 10 m above the seafloor (approx. 4100m).
Data exclusions	No data was excluded from the dataset.
Reproducibility	Code and files for all analyses and graphical interpretations are provided in the supplementary material.
Randomization	The data was collected as part of a oceanographic time series, the fixed stations and depths is part of a pre-established monitoring program of the Northeastern subarctic Pacific Ocean.
Blinding	the data was acquired from an observational sample collection, it was not part of a manipulative study.

Did the study involve field work? ☒ Yes ☐ No

Field work, collection and transport

Field conditions	All samples were collected on board CCGS John P. Tully which is fully equipped for scientific research, operated by the Canadian coast guard and in collaboration with the Institute for Ocean Sciences (IOS), Sidney, Canada which run the scientific sampling program Line P.
Location	station P26 at 50 °N, 145 °W. Water depths: 10m, 25, 50, 100, 150, 200, 300, 400, 600, 800, 1000, 1250, 1500, 2000, 3000, 4000, 10 above seafloor.
Access & import/export	All samples was collected in collaboration with Institute for Ocean Sciences (IOS), Sidney, Canada under their Line P program.
Disturbance	the field sampling caused no disturbance of the site.

Reporting for specific materials, systems and methods

We require information from authors about some types of materials, experimental systems and methods used in many studies. Here, indicate whether each material, system or method listed is relevant to your study. If you are not sure if a list item applies to your research, read the appropriate section before selecting a response.

Materials & experimental systems

n/a	Involved in the study
☒	☐ Antibodies
☒	☐ Eukaryotic cell lines
☒	☐ Palaeontology and archaeology
☐	☒ Animals and other organisms
☒	☐ Human research participants
☒	☐ Clinical data
☒	☐ Dual use research of concern

Methods

n/a	Involved in the study
☒	☐ ChIP-seq
☒	☐ Flow cytometry
☒	☐ MRI-based neuroimaging

Animals and other organisms

Policy information about studies involving animals; ARRIVE guidelines recommended for reporting animal research

Laboratory animals	the study did not involve laboratory animals.
Wild animals	the study did not involve wild animals.
Field-collected samples	seawater was collected, filtered onto 0.2 sterivex filters, and preserved with Sucrose Lysis Buffer before stored at -80 celsius, onboard of the sampling vessel. On land all samples were stored at or below -70 celsius degrees until further processing.
Ethics oversight	No ethical approval was required. The biological material collected from the seawater was marine microbial cells and environmental DNA.

Note that full information on the approval of the study protocol must also be provided in the manuscript.
